# The Impact of Psychological Support on Weight Loss Post Weight Loss Surgery: a Randomised Control Trial

**DOI:** 10.1007/s11695-014-1428-2

**Published:** 2014-09-09

**Authors:** Jane Ogden, Amelia Hollywood, Christopher Pring

**Affiliations:** 1 Department of Psychology, University of Surrey, Guildford, GU2 7XH UK; 2 Western Sussex Hospitals NHS Foundation Trust, St Richard’s Hospital, Spitalfield Lane, Chichester, West Sussex PO19 6SE UK

**Keywords:** Bariatric surgery, Obesity, Weight loss, Psychological support

## Abstract

**Background:**

The purpose of the present study is to evaluate the impact of a health psychology-led bariatric rehabilitation service (BRS) on patient weight loss following bariatric surgery at 1 year.

**Methods:**

A single-site open-randomised parallel group control trial based at St. Richard’s Hospital in Chichester in the UK. Patients (*n* = 162) were recruited immediately prior to Roux-en-Y gastric bypass and randomly allocated to receive either treatment as usual (*n* = 80) or the BRS (*n* = 82). The BRS involved three 50-min one-to-one sessions with a health psychologist and provided information, support and mentoring pre and post surgery addressing psychological issues such as dietary control, self esteem, coping and emotional eating. Weight loss was assessed at 1 year. The key outcome variable was BMI and change in BMI.

**Results:**

Follow-up weight was available for 145 patients. Intention-to-treat analysis (*n* = 162) using last measured weights showed that mean change in BMI by 1 year post surgery was −16.49. There was no significant difference between the two groups (control group = −16.37, 95 % CI = 15.15–17.57; intervention = −16.6, 95 % CI = 15.42–17.81; *η*_p_^2^ = 0.001). Similarly, explanatory analysis (*n* = 145) showed a mean change in BMI of −17.17. The difference between the two groups was not significant (control group = −16.9, 95 % CI = 15.78–18.18; intervention = −17.35, 95 % CI = 18.5–16.16; *η*_p_^2^ = 0.001).

**Conclusions:**

Psychological support pre and post bariatric surgery had no impact on weight loss as measured by BMI and change in BMI by 1 year. It is argued that psychological support should be targeted to patients who start to demonstrate weight regain at a later stage.

Trial registration: ClinicalTrials.gov NCT01264120.

## Introduction

Although weight loss and other associated health outcomes are greater following weight loss surgery (WLS) than those achieved by either medication or behavioural interventions [1–5], such results are not consistent for all patients and a large minority of patients either do not show the desired loss of excess weight or regain weight by follow-up [4, 6–8]. Previous qualitative and quantitative research has explored the mechanisms involved in successful and failed weight loss surgery to explore how effectiveness could be improved [9–11]. The results indicated that whilst successful surgery was associated with a reduction in hunger and preoccupation with food and a sense of being more in control of food intake, less successful surgery was associated with feeling unprepared for the changes required after surgery, reporting being unsupported in the time following surgery and a sense that psychological issues relating to dietary control, self esteem, coping and emotional eating remain neglected. This highlights key areas that need to be addressed to improve patient outcomes following surgery. It also reflects research emphasising the role of psychological factors in predicting outcomes following surgery [12–14]. Further, it confirms the conclusions made by several research teams [15–17] who have argued that WLS patients require multidisciplinary care including psychological input pre and post surgery. Similarly, it reflects recent studies which have offered either lifestyle interventions or counselling pre or post surgery to improve outcomes [18, 19]. Further, both NICE guidelines [20] and those by AACE/TOS/ASMBS [21] state that WLS should be undertaken only by a multidisciplinary team that can provide psychological support before and after surgery. Contrary to these guidelines, however, postoperative psychological support is currently missing in the current standard package of care that is commissioned by NHS England.

The present study therefore aimed to evaluate the impact of a health psychology-led bariatric rehabilitation service (BRS) on patient health outcomes following surgery. The BRS offered information, support and mentoring pre and post surgery and finds reflection in the rehabilitation services which are now a common place for patients post heart attack and stroke [22, 23]. This approach was used as the model for the current intervention as longer-term weight loss success post WLS requires changes in the patients’ cognitions and behaviours relating to diet and exercise and adherence to their new dietary regimen which finds reflection in the behaviour change perspective of rehabilitation programmes for other chronic conditions. It was also developed to reflect the findings of previous qualitative and quantitative research exploring the predictors of failed and successful surgery [9–11]. The present paper reports data for the primary outcome, namely weight loss, by 1 year. Data for the secondary outcomes relating to quality of life, emotional eating and coping are not presented here due to lower response rates for the questionnaire component of the study by 12 months making the study underpowered for these variables.

## Materials/Subjects and Methods

### Participants

St. Richard’s Hospital in Chichester, West Sussex, UK, offers an NHS-based bariatric service for patients with extreme obesity with a BMI over 40 (or 35 with serious co morbidities). Consecutive adult patients were recruited, if they consented, once they had been assessed by the multidisciplinary bariatric team (physician, anaesthetist, dietician, psychologist and surgeon) and approved for surgery. Recruitment took place over a 14-month period from October 2011 to December 2012. Those who could not effectively read or speak English were excluded as this would pose a difficulty in implementing the intervention.

### Design and Procedures

The study involved an open-randomised parallel group control trial with patients allocated to receive either usual care or the bariatric rehabilitation service (BRS) pre and post bariatric surgery. Weight loss was assessed in the clinic at 1-year follow-up. Patients were randomised using third-party-blinded randomization provided by the clinical trial unit at the University of Surrey. Information sheets were sent out in advance of the patients’ preoperative appointment. Two weeks prior to their operation, patients attended the bariatric clinic for a preoperative appointment to have routine tests. At this appointment, patients saw the research fellow who explained the trial, obtained informed consent and randomised them to one of the two arms of the trial. Baseline measures were completed at this time.

### Intervention

Patients allocated to the usual care (control) group received preoperative tests and a standard diet sheet postoperatively informing them about their desired diet and the stages of food progression from only consuming liquids to soft food then back to all foods. Patients returned for surgery approximately 2 weeks later, and after a median post surgical stay of two nights, they were discharged home. They then returned to the clinic at 6 weeks, 3, 6 and 12 months to see the dietician and/or specialist nurse.

Patients allocated to the BRS (intervention) group received usual care as described above plus three one-to-one 50-min sessions with a health psychologist 2 weeks preoperatively, postoperatively (before they were discharged from hospital) and at 3 months follow-up. The design of the BRS was based on the preparation procedures for surgery and cardiac rehabilitation services used for patients post MI [22, 23]. The programme was also developed in line with the needs of bariatric patients following previous qualitative and quantitative research [9–11] and ongoing input from users of two active support groups who had highlighted the need for increased psychological input. The health psychologist used both didactic and non didactic methods and addressed five key factors: (i) knowledge (i.e. information about dietary change), (ii) beliefs (concerning the causes and solutions to obesity), (iii) behaviours (with a focus on diet and physical activity), (iv) coping strategies (i.e. managing emotions without using food, identifying alternative and healthy methods of coping, managing other addictions) and (v) adjustment (i.e. exploring ways to work with the restriction imposed by the operation). Details concerning the structure of the sessions can be found in the protocol [24].

### Primary Outcome Measure

Patients’ weight was obtained in the clinic 2 weeks preoperatively and postoperatively at 3, 6 and 12 months. The primary outcome was weight loss at 12 months measured in terms of BMI and change in BMI. Participant demographics (age, sex, ethnic group, education, living status, years trying to lose weight) were also assessed at baseline.

### Statistical Analysis

Initially, differences between the conditions for baseline demographics were assessed using *χ* ^2^ and *t* tests. Differences in weight loss outcomes were assessed in terms of BMI at baseline and 12 months follow-up and change in BMI using *t* tests.

## Results

### Flow of Participants, Follow-up and Sample Characteristics

**Fig. 1:**
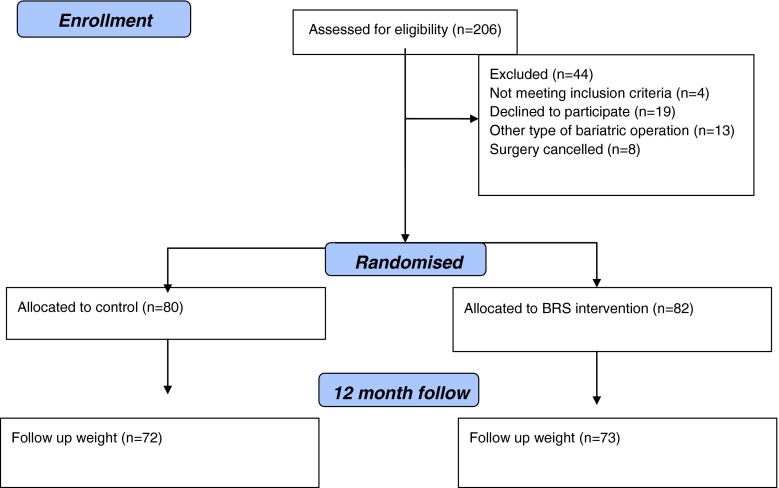
CONSORT flow diagram

Two hundred six patients were invited to take part in the study, 19 refused and 184 consented (see Fig. 1). Of these 9 had a gastric band, 4 had a sleeve gastrectomy and 8 had their operation cancelled and so were excluded from the analysis. The remainder received a Roux-en-Y gastric bypass (*n* = 162) (response rate = 79.8 %) and were allocated to either the usual care arm (*n* = 80) or the BRS intervention (*n* = 82). Follow-up weights at 12 months were obtained from *n* = 145 (usual care *n* = 72; BRS *n* = 73). Seventeen weights were unavailable as patients did not return for their 1-year appointment at the clinic. For the intention-to-treat analysis, these final weights were imputed from either their weight at baseline (*n* = 2), at 3 months follow-up (*n* = 4) or 6 months follow-up (*n* = 11). The subsequent explanatory analysis was based upon *n* = 145.

Participants’ baseline demographics (*n* = 162) for all patients and by trial arm are shown in Table 1. Mean weight at baseline was 142.85 kg (range 96.5–250.8 kg), mean BMI was 50.65 (SD = 7.81) and the majority were white, female, cohabiting, educated to either secondary school or the level of a professional certificate with a mean age of 45 years. The results showed no differences between the two arms in terms of demographics.

**Table 1:** Participant demographics (total sample and by trial arm)

		All participants (*n* = 162)	Usual care control (*n* = 80)	BRS intervention (*n* = 82)	*χ* ^2^/*F*/*p*
Age	Mean (SD)	45.2 (10.84)	44.8 (10.6)	45.6 (11.11)	*t* = −0.4 *p* = 0.7
	Range	18–68	18–66	21–68	
Sex	M	40 (24.7 %)	19 (23.8 %)	21 (25.6 %)	*χ* ^2^ = 0.07 *p* = 0.7
	F	122 (75.3 %)	61 (76.2 %)	61 (74.4 %)	
Ethnic group	White	156 (96.4 %)			
	Black	2 (1.2 %)			
	Asian	2 (1.2 %)			
	Other	2 (1.2 %)			
Education	<Sec. school	5 (3.1 %)	2 (2.5 %)	3 (3.7 %)	*χ* ^2^ = 4.19 *p* = 0.4
	Sec. school	74 (46 %)	33 (41.8 %)	41 (50.0 %)	
	Prof cert.	59 (36.6 %)	35 (44.3 %)	24 (29.3 %)	
	Degree	20 (12.4 %)	8 (10.1 %)	12 (14.6 %)	
	Higher degree	3 (1.9 %)	1 (1.3 %)	2 (2.4 %)	
Employ	FT	60 (37.0 %)	28 (35.0 %)	32 (39.0 %)	*χ* ^2^ = 1.1 *p* = 0.58
	PT	35 (21.6 %)	20 (25.0 %)	15 (18.3 %)	
	Neither	67 (41.4 %)	32 (40 %)	35 (42.7 %)	
Baseline BMI	Mean (SD)	50.65 (7.81)	50.89 (8.33)	50.42 (7.31)	*t* = 0.38 *p* = 0.7
	Range	36.1–74.75	36.1–74.8	38.7–69.5	
Baseline weight	Mean (SD)	142.85 (27.0)	140.85 (27.0)	143.79 (29.2)	*t* = −0.66 *p* = 0.51
	Range	96.5–250.8	CI = 134–147.02	CI = 137–149.9	
Living status	Alone	27 (16.7 %)	17 (21.2 %)	10 (12.2 %)	*χ* ^2^ = 2.39 *p* = 0.12
	Cohabiting	135 (83.3 %)	63 (78.8 %)	72 (87.8 %)	
Years trying to lose weight	Mean (SD)	23.57 (12.31)	22.51 (12.13)	24.61 (12.46)	*t* = −1.07 *p* = 0.29
	Range	0–60	3–55	0–60	

### Impact of the BRS on Weight Loss by 1-Year Follow-up

#### Intention-to-Treat Analysis

Following imputation of missing final weight, the mean BMI at follow-up was 34.2 (SD = 6.1) ranging from 21.4 to 51.04. No difference was found between the usual care group (34.53 (SD = 6.4; 95 % CI = 33.17–35.88)) or the BRS group (33.8 (SD = 5.86; 95 % CI = 32.48–35.14)), (*t*(160) = 0.7, *p* = 0.5, *η*_p_^2^ = 0.003). In terms of change in BMI for all participants (*n* = 162), the mean BMI change by 1 year was −16.48 (SD = 5.48). Those in the usual care group showed a mean change in BMI of −16.37 (SD = 5.6; 95 % CI = 15.15–17.57) and those in the BRS intervention group showed a mean change in BMI of −16.6 (SD = 5.4; 95 % CI = 15.42–17.81). This difference was not significant (*t*(160) = 0.3, *p* = 0.7, *η*_p_^2^ = 0.001). The mean weight loss by 12 months was −46.37 kg with no differences between the groups (control 45.28 kg; intervention 47.45 kg; *η*_p_^2^ = 0.004).

#### Explanatory Analysis

The mean BMI for all participants with complete data at 12 months (*n* = 145) was 33.6 (SD = 5.94) ranging from 21.4 to 50.37. Those in the usual care group (*n* = 72) showed a mean final BMI of 34.1 (SD = 6.1; 95 % CI = 32.7–35.5) and those in the BRS intervention group (*n* = 73) showed a mean BMI of 33.19 (SD = 5.76; 95 % CI = 31.8–34.6). This difference was not significant (*t*(143) = 0.9, *p* = 0.4; *η*_p_^2^ = 0.006). Finally, in terms of change in BMI at follow-up, the mean change in BMI for participants with complete weight data by 12 months was −17.17 (SD = 5.13). No difference in BMI change was found between the usual care group (−16.9 (SD = 5.2; 95 % CI = 15.78–18.18)) and the BRS group (−17.35 (SD = 5.1; 95 % CI = 16.16–18.5); (*t*(143) = 0.4, *p* = 0.7, *η*_p_^2^ = 0.001)). Using this analysis, the mean weight loss was 48.37 kg with no differences between the groups (control 47.11 kg; intervention, 49.62 kg; *η*_p_^2^ = 0.006).

## Discussion

Current NICE and AACE/TOS/ASMBS guidelines [20, 21] promote psychological support both pre and post surgery and several research groups [15–19] have argued that bariatric patients require multidisciplinary care including psychological input. The present study therefore aimed to provide an evidence base for psychological support pre and post bariatric surgery as a means to promote weight loss by 1 year.

The results showed that at 12 months follow-up, the health psychology-led bariatric rehabilitation service had no impact on weight loss in terms of BMI and change in BMI, with patients showing a mean change in BMI of −16.49 regardless of group (control group = −16.37 versus BRS group = −16.6). Furthermore, no differences were found in terms of absolute BMI by follow-up (control group = 34.53 versus BRS group = 33.8) with these analyses being comparable for both the intention-to-treat and explanatory analyses.

These findings clearly indicate that psychological support pre and post bariatric surgery does not significantly influence weight loss and could be used to indicate that such psychological support is unnecessary and should be removed from NICE guidelines. There are several issues with the study, however, that need to be considered. First, follow-up weight was measured at 1 year post surgery rather than in the longer term. Research indicates that weight loss variability is most evident between 18 and 24 months post surgery [4, 8]. Perhaps the benefits of psychological support as delivered in the current trial could be seen at a later follow-up. Furthermore, the intervention may be effective at preventing weight regain rather than weight loss per se which requires a longer-term follow-up. Second, the present paper presents the results in terms of our primary outcome variable, namely weight loss. It is possible that the secondary psychological variables will show a greater difference between the two groups. The equivalent findings from the present study, however, may have less to do with the timing of the follow-up or the type of outcomes being assessed but the timing of the intervention itself. Due to recent changes in clinical practice, all patients in the present study were given a Roux-en-Y gastric bypass as gastric bands were deemed less suitable for the patient demographic by the onset of recruitment. The gastric bypass produces fairly consistent changes in weight by 1 year leaving very little variability to be addressed by a psychological intervention. Accordingly, the first-year post surgery is characterised by a dramatic change in what can be consumed, the need to cope with side effects such as discomfort, pain and dumping and a gradual adjustment to the required changes in diet. The first year therefore is best characterised as a time when the surgery has a clear direct effect rather than being a tool to be worked with [9]. Psychological support may therefore be more effective if delivered after this first year, when evidence shows increased variability in weight change and when issues such as emotional eating and coping become more of an issue and changes in eating behaviour return to being more under the volition of the individual. Similarly, psychological input may best be reserved in a targeted manner rather than being applicable to every patient in the postoperative setting.

To conclude, psychological support immediately pre and post surgery was found to have no effect on weight loss at 1 year. Further research is required to evaluate the longer-term implications for both weight loss and psychological variables and to explore alternative, more effective timings for such an intervention.
